# The Zebrafish Sperm Proteome

**DOI:** 10.1002/pmic.202400310

**Published:** 2024-12-31

**Authors:** Jayme Cohen‐Krais, Carlo Martins, Jamie Bartram, Zoe Crighton, Jean‐Charles de Coriolis, Alice Godden, Daniel Marcu, Weronika Robak, Gerhard Saalbach, Simone Immler

**Affiliations:** ^1^ School of Biological Sciences University of East Anglia Norwich Research Park Norwich Norfolk UK; ^2^ Biochemistry & Metabolism, John Innes Centre Norwich Research Park Norwich Norfolk UK

**Keywords:** *Danio rerio*, fertilisation, fertility, proteomics, sperm function, sperm‐egg interaction

## Abstract

One of the key processes that forms the basis of fertilisation is the tight interaction between sperm and egg. Both sperm and egg proteomes are known to evolve and diverge rapidly even between closely related species. Understanding the sperm proteome therefore provides key insights into the proteins that underpin the mechanisms involved during fertilisation and the fusion between sperm and egg, and how they can differ across individuals of the same species. Despite being a commonly used model organism for reproductive research, little is currently understood about the zebrafish *Danio rerio* sperm proteome. We performed nanoLC‐MS/MS proteomics analysis after off‐line sample fractionation with six pooled samples containing sperm from ten males each. We confidently identified 5410 proteins, from which a total of 3900 GeneIDs were generated leading to 1720 Gene Ontology terms.

1

Sperm are highly complex cells with key functions to ensure reproduction and fertility. The proteome of sperm reveals information about sperm function including motility, capacitation and fertilisation. Sperm morphology (and therefore the proteome) shows significant variation across species and individuals, but also between ejaculates from the same individual due to causes such as age, disease, environmental conditions and other health‐related factors [1]. The identification and quantification of the proteins present in sperm help to pinpoint potential biomarkers associated with male reproduction and fertility.

According to Web of Science (Clarivate (Web of Science). Clarivate 2024. All rights reserved.), “sperm proteomes” have been described in 891 papers, including 34 human papers and 857 papers on other species. The current total number of proteins identified in the human sperm proteome is 6871 combined across all accessible human sperm proteomics datasets, with a maximum of 5685 identified from a single experiment [2]. Similarly, in the domestic bull *Bos taurus*, proteomic analyses of high and low motility sperm populations revealed that 498 proteins differed between high and low fertility bulls [3]. Furthermore, the number of proteins identified in sperm proteomes differ by up to 21.6% across three mouse species [4, 5, 6, 7], exhibiting divergent mating systems thought to drive the evolution in the sperm proteome. In the oyster *Crassostrea hongkongensis*, the sperm proteome was used to establish the molecular mechanisms underlying species‐specific sperm and oocyte binding in free‐spawning organisms [8], and similarly, high levels of variation in the sperm proteome have been identified across marine mussel species *Mytilus edulis* and *Mytilus galloprovincialis* [9]. These previous studies highlight the diversity and rapid divergence of sperm proteomes even between closely related species as an indicator of its key role in reproduction. In teleost fish species such as the rainbow trout *Oncorhynchus mykiss*, 206 proteins have been identified from sperm [10], whereas in carp *Cyprinus carpio* L, 348 proteins were identified [11]. This variation in protein numbers is likely not only due to biological differences between species but also the analytical methods used to determine the proteomes. More accurate data are therefore needed to allow further research into the role of the sperm proteome in sperm function and sperm‐egg interactions.

The zebrafish *Danio rerio* is a popular model organism for biomedical research ranging from ecotoxicology, evolutionary and developmental biology, cancer and neurodegenerative diseases, and ageing [12]. As an externally fertilising vertebrate, collection of gametes and in vitro fertilisation is particularly easy and can be performed mimicking natural conditions, rendering the zebrafish a prime model organism to study fertility and reproduction [13]. Proteomic profiles have been developed for a wide range of zebrafish tissues including the testes (2214 described proteins) and the ovaries (1379 described proteins) [14]. In addition, the zebrafish oocyte proteome has been described with 1568 proteins differing between mature and immature oocytes [15] and the proteome profiling of zebrafish embryos has been used to study the effects of environmental stressors such as toxins [16]. The available proteomic profile of the zebrafish currently includes 53,918 proteins on the NCBI Refseq protein database [17]. However, despite the proliferation of proteomic profiling of zebrafish tissues and cell types, the global proteomic profiling of zebrafish sperm is currently lacking. Here, the global proteomic profile of pooled zebrafish sperm was obtained through tandem liquid chromatography‐mass spectrometry (LC‐MS/MS) and analysed through the comparison of relative peptide abundance as well as Gene Ontology.

Sperm were collected from sexually mature ABWT (AB Wild Type strain) male zebrafish, originally obtained from the Zebrafish International Resource Center (ZIRC, Oregon) and maintained at the Controlled Environment Facility at the University of East Anglia, UK. The males were between 1 and 1.5 years old and at the peak of reproductive age. Prior to gamete collection, the fish were maintained in 3.5 L tanks at a 10:6 male to female sex ratio kept at 28°C (standard temperature) or 34°C (high temperature) to test for a potential role of environmental factors shaping the sperm proteome with a 14:10 light schedule and fed ad libitum with a mix of dry feed (ZEBRAFEED 400–600 by SPAROS, Área Empresarial de Marim, Lote C, 8700‐221 Olhão, Portugal) and live *Artemia* (Sep‐Art Artemia Cysts, Ocean Nutrition). All fish used for experiments were kept under Home Office license P0C37E901. Following anaesthesia using metomidate hydrochloride (Aquacalm by Syndel, #9, 4131 Mostar Rd, Nanaimo, BC V9T 6A6 Canada), males were stripped for sperm through gentle squeezing in a cranio‐caudal direction and the ejaculates were collected immediately into a microcapillary tube. Due to the low number of cells produced in a single zebrafish ejaculate, sperm samples from ten males were pooled into one replicate sample to ensure sufficient material for mass spectrometry, and we collected a total of six biological replicates (60 males). These sperm pools were washed in Hanks’ Balanced Salt Solution to remove seminal fluid, and pelleted through centrifugation for 8 min at 8000 rpm.

Following washing, sperm pool pellets were precipitated in acetone (50 µL + 900 µL, 10 mM NaCl) under vortexing on ice for 2 h. The pellets were then resuspended in 50 µL of SDC buffer (2.5% sodium deoxycholate (SDC; Merck) in 0.2 M EPPS‐buffer (Merck), pH 8.5) and vortexed under heating. Aiming at 50 µg protein per sample, estimated by a Direct Detect fluorometric assay (MERCK MILLIPORE, Dorset, UK), equal amounts of protein per sample were reduced, alkylated, and digested with trypsin in the SDC buffer according to standard procedures adapted from Shevchenko et al. [18]. After the digest, the SDC was precipitated by adding an equal volume of 0.4% TFA, and the clear supernatant subjected to C18 SPE (OMIX tips; Agilent). Peptide concentration was further estimated by analysing a small aliquot of the digests by LCMS, as described below, to estimate the total peptide abundance per sample. Sample aliquots corresponding to estimated equal peptide abundances were used for TMT labelling using a TMT 6plex kit (Lot VB290974, ThermoFisher Scientific, Hemel Hempstead, UK) according to the manufacturer's instructions with slight modifications. However, this labelling was not used for quantification and is not relevant for the analysis presented in this paper. Six differently labelled samples were pooled and desalted using a C18 Sep‐Pak cartridge (200 mg, Waters, Wilmslow, UK). The eluted peptides were dissolved in 500 µL of 25 mM NH_4_HCO_3_ and fractionated by high pH reversed phase HPLC. For this, the samples were loaded to an XBridge 3.5 µm C18 column (150 × 3.0 mm, Waters). Fractionation was performed on an ACQUITY Arc Bio System (Waters) with the following gradient of solvents A (water), B (acetonitrile), and C (25 mM NH_4_HCO_3_ in water) at a flow rate of 0.5 mL/min: solvent C was kept at 10% throughout the gradient; solvent B: 0–5 min: 5%, 5–10 min: 5%–10%, 10–60 min: 10%–40%, 60–75 min: 40%–80%, followed by 5 min at 80% B and re‐equilibration to 5% for 24 min. Fractions of 0.5 mL were collected every 1 min and concatenated by combining fractions of similar peptide concentration to produce 19 final fractions of the sperm samples for MS analysis. Aliquots were analysed by nanoLC‐MS/MS on an Orbitrap Eclipse Tribrid mass spectrometer coupled to an UltiMate 3000 RSLCnano LC system (Thermo Fisher Scientific, Hemel Hempstead, UK). The samples were loaded onto a trap cartridge (Pepmap 100, C18, 5um, 0.3 × 5 mm, Thermo) with 0.1% TFA at 15 µL/min for 3 min. The trap column was then switched in‐line with the analytical column (nanoEase M/Z column, HSS C18 T3, 1.8 µm, 100 Å, 250 mm × 0.75 µm, Waters) for separation using the following gradient of solvents A (water, 0.1% formic acid) and B (80% acetonitrile, 0.1% formic acid) at a flow rate of 0.2 µL/min: 0–3 min 3% B (parallel to trapping); 3–10 min linear increase B to 8%; 10–75 min increase B to 37%; 75–90 min linear increase B to 50%; followed by a ramp to 99% B and re‐equilibration to 3% B. Data were acquired with the following parameters in positive ion mode: MS1/OT: resolution 120K, profile mode, mass range *m*/*z* 400–1800, AGC target 100%, max inject time 50 ms; MS2/IT: data dependent analysis with the following parameters: top10 in IT Turbo mode, centroid mode, quadrupole isolation window 0.7 Da, charge states 2–5, threshold 1.9e4, CID CE = 35, AGC target 1e4, max. inject time 70 ms, dynamic exclusion 1 count for 15 s mass tolerance of 7 ppm; MS3 synchronous precursor selection (SPS): 10 SPS precursors, isolation window 0.7 Da, HCD fragmentation with CE = 65, Orbitrap Turbo TMT and TMTpro resolution 30k, AGC target 1e5, max inject time 105 ms, Real Time Search (RTS): protein database *D. rerio* (Uniprot reference proteome UP000000437, August 2022, 46,842 entries) *,* enzyme trypsin, 1 missed cleavage, oxidation (M) as variable, carbamidomethyl (C) and TMT as fixed modifications, Xcorr = 1, dCn = 0.05. The LCMS analysis was done in 2022 using the database available at that time. The final processing reported here was done in 2024 using the updated database from 2024.

The acquired raw data from the 19 HPLC fractions were processed and quantified using peptide peak intensities in Proteome Discoverer 3.1 (Thermo); all mentioned tools of the following workflows are nodes of the proprietary Proteome Discoverer (PD) software. The *D. rerio* fasta database (Uniprot UP000000437_Danio_7955.fasta, April 2024, 25,991 entries) was imported into PD adding a reversed sequence database for decoy searches. The processing workflow included Minora Feature Detection with min. trace length 7, S/N 5, PSM confidence high, and Top N Peak Filter with 20 peaks per 100 Da. For the database search the CHIMERYS search engine (MSAID, Munich, Germany) was used with the inferys_3.0.0_fragmentation prediction model with FDR targets 0.01 (strict) and 0.05 (relaxed), a fragment tolerance of 0.3 Da, enzyme trypsin with one missed cleavage, variable modification oxidation (M), fixed modifications carbamidomethyl (C), and TMT 6plex on N‐terminus and K.

The consensus workflow was set up to quantify peptides and proteins based on their ion count intensity combined from all HPLC fractions of the six pooled samples. For identification, an FDR of 0.01 was used as strict threshold. Protein abundance was calculated using the top three most abundant peptides unique to protein groups. The results were exported into a Microsoft Excel table including data for protein abundances, number of peptides, protein coverage, the search identification score and other important values.

Processed data were then further analysed and visualised using R version 4.0.3 (The R Foundation for Statistical Computing, Vienna, Austria, URL https://www.R‐project.org/). ID mapping was performed using Uniprot [19] as well as the STRING database [20]. Gene Ontology (GO) term analysis was performed using ShinyGO 0.76.2 [21].

We found no significantly differentially expressed proteins between sperm from males kept at 28°C or 34°C assuming a significance cutoff of p_adj_ < 0.05, and hence pooled all data for further interpretation. We found substantial variation across the three biological sample pools from males kept at 34°C, which may explain the lack of significantly differentially expressed proteins. Higher sample sizes may help identify more subtle differences in sperm proteomes across temperature treatments and future studies are needed to address this question in more detail. The proteome produced for the combined six pooled sperm samples included 5410 proteins after filtering to remove single peptide matches and potential contaminants (see Table S1 in Supporting Information for full list). Of these 5410 proteins, 4168 could be quantified, while 1242 could not be. Compared to the proteomes in rainbow trout [10] and carp [11], the number of proteins identified in zebrafish sperm described here is considerably higher and likely a result of the different methods used.

The Uniprot UP000000437_Danio_7955.fasta reference proteome of the zebrafish used in this study contains one protein per gene (25,991 entries) [19], The GRCz11 reference genome [22] for zebrafish includes a total of 25,545 coding genes. In our study, 5410 proteins were detected, which suggests that roughly 20% of the potential zebrafish gene products were detected. Comparatively, proteomic profiles of other zebrafish organs identified ∼1300 proteins in muscle tissue (5% of gene products), ∼1500 proteins in skin tissue (6% of gene products) and ∼2400 proteins in gill tissue (9% of gene products) resulting in a total of over 5000 proteins across all tissues [23].

The top 25 most abundant proteins from the sample pools include eight different histone related proteins including Histone H3‐like (*A0A8M6Z1M4)*, H1.0 linker histone (*Q6NYV3*), Histone H2B (*X1WHF1*),Histone H1‐like (*A0A8M6Z7Z5*), Histone H1‐like (*Q568D0*), Histone H2A (*Q0Z946*), Histone H2AX (*Q7ZUY3*), and Core histone macro‐H2A (*Q4V914*). The high abundance of histones in zebrafish sperm is to be expected due to the lack of protamine‐replacement and maintenance of histone packaging during sperm chromatin condensation during spermatogenesis [24]. The top 25 proteins were also mapped with the STRING database [20] (Figure 1), and while 11 of these could not be identified, the largest continuous network determined by this database included five of the histone related proteins mentioned above as well as the alpha (*Q08BA1*) and beta (*A8WGC6*) subunits of ATP‐synthase and heat shock protein HSP 90‐ beta (*O57521*).

**FIGURE 1:**
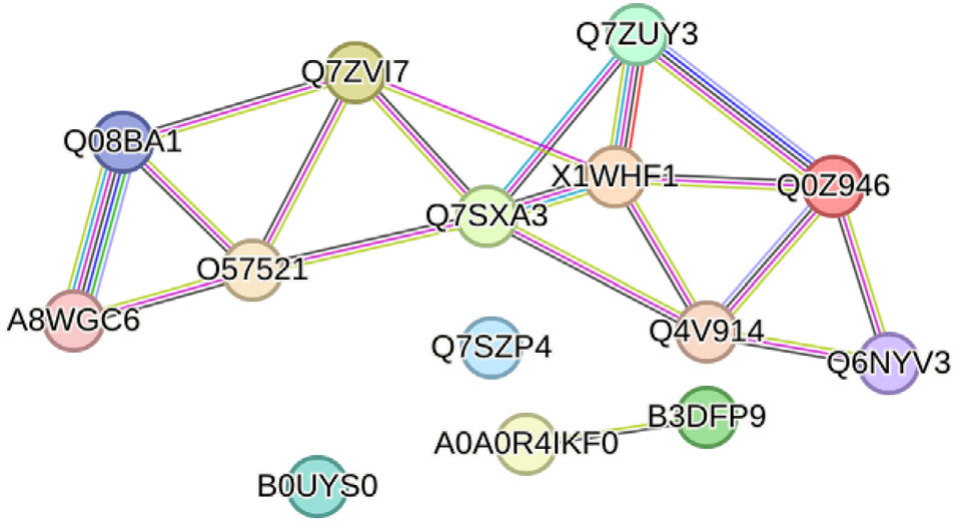
The STRING database map generated through the input of the protein accession ID of the 25 most abundant proteins detected in this data.

According to the Uniprot ID database [19], of the 5410 proteins that were confidently identified in this dataset, 3449 (63.8%) were listed as having previously been identified with evidence at the protein level, 1042 (19.3%) were found at the transcript level, 473 (8.7%) were inferred from homology, and 446 (8.2%) were predicted from the zebrafish genome but have no previously recorded protein evidence. These 5410 proteins were matched to 4844 NCBI GeneIDs using the Uniprot ID mapping software. A total of 566 proteins could not be assigned NCBI GeneIDs.

Using the assigned GeneIDs, a total of 1919 GO terms were identified among the three categories of GO terms, including 395 cellular components (Figure 2A), 1001 biological processes (Figure 2B), and 523 molecular functions (Figure 2C). Two of the most fold‐enriched cellular component GO terms were the endopeptidase and peptidase complexes. Both the endopeptidase and peptidase complex have been previously noted as playing an integral role in sperm motility due to the potential for these proteins to act as intermediates in protein–protein networks in response to stimuli [25]. Of the top 20 cellular component GO terms, six were mitochondria related. This is to be expected as mitochondria play an important role in the energy production required for flagellum movement and therefore sperm motility [26]. Mitochondria have also previously been noted as undergoing ultrastructural changes in zebrafish sperm in response to activation and sperm motility [27]. The biological processes included in the top 20 GO terms by fold‐enrichment include a number of biosynthetic processes for molecules such as peptides and amides as well as the establishment of protein localisation. Since this proteome was established from mature sperm, these GO terms suggest that proteins that are integral to these processes during spermatogenesis may be retained in the mature sperm, but listed functions may have change in activity—an aspect that needs further testing. The highest fold‐enrichment molecular function GO term was ligase activity, and nine of the top 20 molecular functions were molecular binding terms such as anion binding and small molecule binding. These terms may indicate the importance of energy production and anion binding, which play integral roles for zebrafish sperm activation in response to osmotic pressure via a hypo‐osmotic shock caused by the decrease in extra‐cellular potassium upon contact with water after ejaculation to activate flagellar motility [27, 28].

**FIGURE 2:**
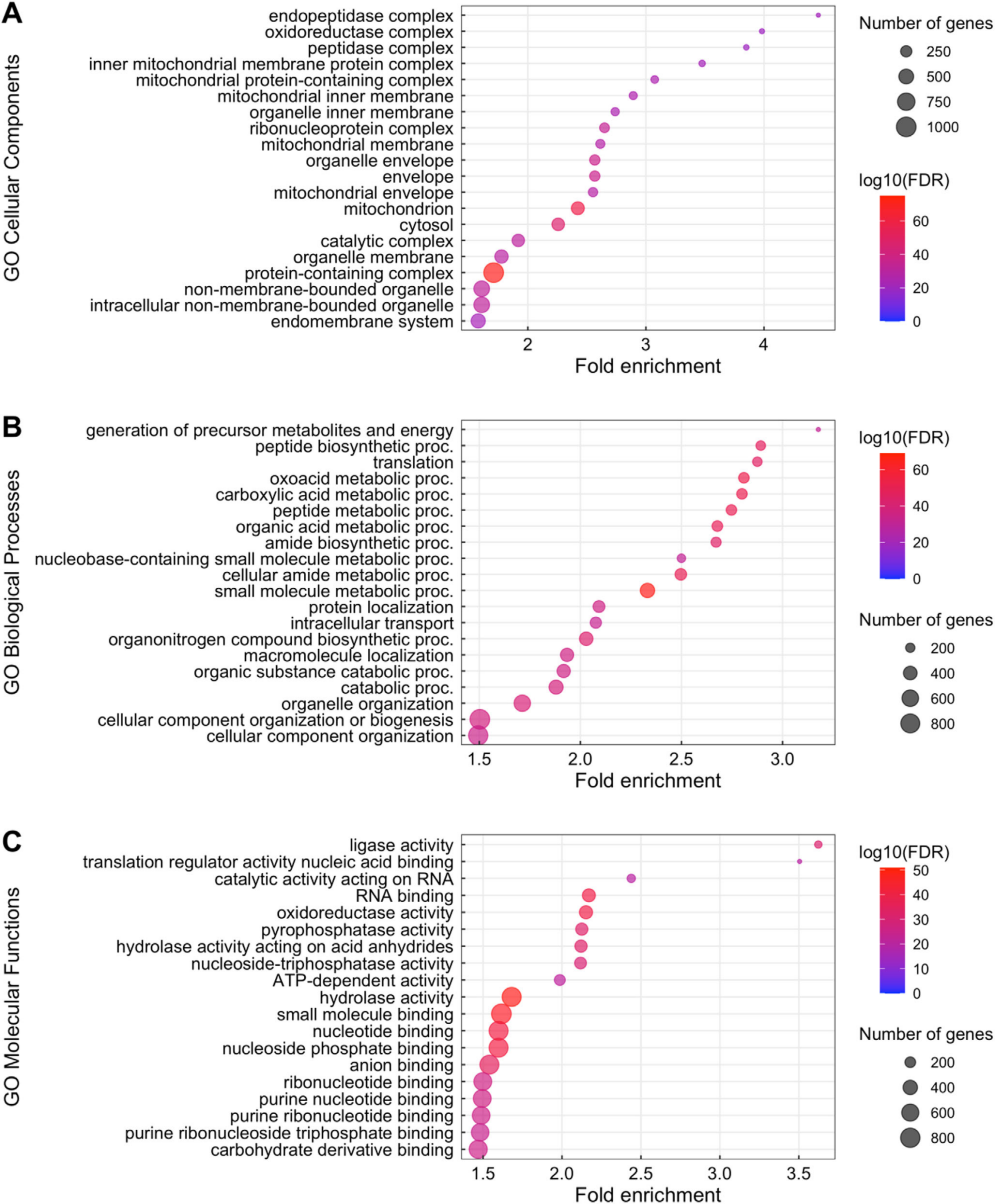
Top 20 GO terms ranked by fold‐enrichment for the zebrafish sperm proteome for (A) cellular component GO terms, (B) biological process GO terms and (C) molecular function GO terms for the sperm proteome.

In conclusion, the quantitative mass spectrometry proteomic profiling of zebrafish sperm yielded a proteome list of 5410 proteins. Amongst these proteins, we found a large number of histones, which demonstrates the particular chromatin architecture characterising zebrafish sperm. Many of the other proteins identified in this study reveal interesting details about how many proteins essential to spermatogenesis are retained by mature sperm, due to the most highly ranked biological processes GO terms. The cellular component and molecular functions GO terms reveal the fold‐enrichment of proteins integral to sperm motility and activation. Our data provide a basis and resource for further protein‐level analysis of zebrafish sperm enabling an expansion of this area of study in a commonly used model organism for reproductive research.

## Conflicts of Interest

The authors declare no conflicts of interest.

## Supporting information



Supporting Information

## Data Availability

The mass spectrometry proteomics data have been deposited to the ProteomeXchange Consortium via the PRIDE [29] partner repository with the dataset identifier PXD056057 and https://doi.org/10.6019/PXD056057.
